# A Waterproof Flexible Paper-Based Thermoelectric Generator for Humidity and Underwater Environments

**DOI:** 10.3390/ma17102338

**Published:** 2024-05-14

**Authors:** Yiduo Huang, Wenfeng Wang, Sijia Chang, Aida Bao, Yuan Liu, Ruirui Li, Jijun Xiong

**Affiliations:** 1 State Key Laboratory of Dynamic Measurement Technology, North University of China, Taiyuan 030051, China; huangyiduoz@163.com (Y.H.); 15383466221@163.com (W.W.); csj15035900484@163.com (S.C.); baoaida@126.com (A.B.); xiongjijun@nuc.edu.cn (J.X.); 2 Shenzhen Institute of Advanced Technology, Chinese Academy of Sciences, 1068 Xueyuan Avenue, Shenzhen 518055, China

**Keywords:** flexible thermoelectric generator, modified paper, superhydrophobic layer encapsulation, humidity environment, underwater environment

## Abstract

A thermoelectric generator (TEG) is one of the important energy harvesting sources for wearable electronic devices, which converts waste heat into electrical energy without any external stimuli, such as light or mechanical motion. However, the poor flexibility of traditional TEGs (e.g., Si-based TE devices) causes the limitations in practical applications. Flexible paper substrates are becoming increasingly attractive in wearable electronic technology owing to their usability, environmental friendliness (disposable, biodegradable, and renewable materials), and foldability. The high water-absorbing quality of paper restricts its scope of application due to water failure. Therefore, we propose a high-performance flexible waterproof paper-based thermoelectric generator (WPTEG). A modification method that infiltrates TE materials into cellulose paper through vacuum filtration is used to prepare the TE modules. By connecting the TE-modified paper with Al tape, as well as a superhydrophobic layer encapsulation, the WPTEG is fabricated. The WPTEG with three P–N modules can generate an output voltage of up to 235 mV at a temperature difference of 50 K, which can provide power to portable electronic devices such as diodes, clocks, and calculators in hot water. With the waterproof property, the WPTEG paves the way for achieving multi-scenario applications in humid environments on human skin.

## 1. Introduction

Wearable electronics are growing rapidly because of emerging applications in different scenarios, especially in human health monitoring, intelligent robots, and human–machine interaction [1,2,3]. As an energy source, conventional power modules have serious defects such as frequent charging, replacement, and maintenance. For example, micro batteries with limited power cannot provide long-lasting energy for wearable electronic devices, and the environmental pollution and unexpected explosions also need to be addressed [4,5].

Flexible generators with wearability, sustainability, and eco-friendliness can effectively convert various ambient energies to electricity. At present, the common power supply devices used for energy collection and conversion include flexible solar cells [6,7], piezoelectric generators [8,9], triboelectric nanogenerators, and thermoelectric generators (TEGs) [10,11,12,13]. Solar energy is the most abundant renewable resource, but solar cells are subjected to weather conditions in practical applications. Piezoelectric generators and triboelectric nanogenerators often require continuous motions of the human body. As a constant temperature heat source, the human body can continuously provide a constant temperature for TEGs. TEGs can collect low-grade heat through the temperature difference between the human body and the surrounding environment [14,15]. In order to develop flexible TEGs, various types of substrates (e.g., textiles, PDMS, and hydrogels) have been proposed [16,17,18]. Paper-based flexible thermoelectric generators (PTEGs) have attracted great interest due to their flexibility, low cost, abundant resources, biocompatibility, and environmental friendliness. For example, Li et al. designed a paper-based TEG based on multi-walled carbon nanotubes/carboxylated nanocellulose, which has excellent mechanical flexibility and thermoelectric performance [19]. Kim et al. developed a foldable TEG based on the solution-processed carbon nanotube buckypapers with high power generation efficiency and high-level integration [20]. These paper-based TEGs have high flexibility and efficient energy collection. However, once applied to the skin for a long time, paper-based TEGs often experience power generation performance degradation owing to the inevitable sweaty penetration. Therefore, there is an urgent need to address the issue of unavoidable liquid effects. Many additional materials (e.g., PDMS, PI, and PTFE) are proposed to package paper devices [21,22,23]. Nevertheless, this packaging can also restrain the thermoelectric performance of the devices to a certain extent. Consequently, it is necessary to develop more efficient preparation techniques to solve the problems of sweaty penetration and water failure for paper-based flexible thermoelectric generators.

**Figure 1:**
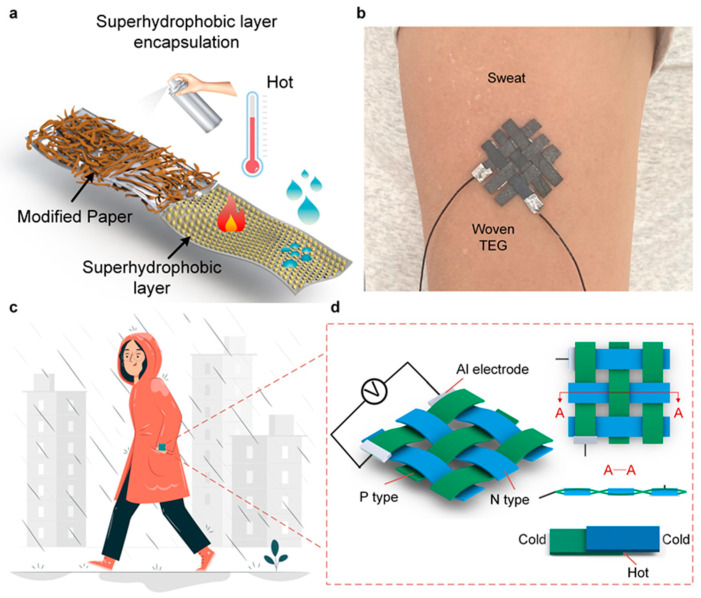
(**a**) Structure diagram of WPTEG and the anti-environment disturbance ability in high temperature and humidity environments. (**b**) Picture of a woven WPTEG applied on a sweating arm. (**c**) A wearable WPTEG in a rainy environment. (**d**) Schematic of the woven structure of WPTEG.

Here, we proposed a waterproof paper-based thermoelectric generator (WPTEG) with high power generation performance, excellent stability, and water resistance. Such a device is fabricated by infiltrating thermoelectric materials (Bi_2_Te_3_ doped with Se and Sb_2_Te_3_ doped with Bi) into a cellulose paper matrix through vacuum filtration. After a superhydrophobic layer encapsulation, it possesses excellent resistance to water permeability as well as environmental disturbances, as shown in Figure 1a. In addition, the water resistance property enables the device to operate in various operational environments, such as a sweating body or wet weather, as depicted in Figure 1b,c. Moreover, the device composed of strip-shaped TE papers and an Al electrode can form a woven structure to apply to the human body (Figure 1d) [24]. The obtained WPTEG with three P–N modules can generate 235.76 mV at a temperature difference of 50 K. Furthermore, owing to the superhydrophobic layer encapsulation, the device can provide stable power for various portable electronics in a water environment. Constructing a waterproofing thermoelectric generator based on cellulose paper substrate provides a facile and practical approach to solving the failure problem of thermoelectric generators when in contact with water and sweat.

## 2. Experimental Section

### 2.1. Materials

Se (powder, ≥99.99%), Bi_2_Te_3_ (powder, 99.99%), Bi (powder, 99.99%), Sb_2_Te_3_ (powder, 99.96%), and Carboxymethylcellulose sodium (CMC-Na) (viscosity 3000–5000 mpa·s) were purchased from Macklin (Shanghai, China). The medium speed qualitative filter papers with a diameter of 9 cm were purchased from Aladdin (Shanghai, China). Al tape was purchased from Youyigu E-Commerce Co., Ltd. (Tianjin, China). The superhydrophobic coating was purchased from Rust-Oleum Corporation (Vernon Hills, IL, USA).

### 2.2. Preparation of TE-Modified Papers

The N–type material is Bi_2_Te_3_ doped with Se, while the P–type material is Sb_2_Te_3_ doped with Bi. Firstly, N–type TE powder and Carboxymethylcellulose sodium (CMC-Na) were dispersed in 30 mL of deionized water at a mass ratio of 12:1, and then the mixture was stirred for 15 min to form the N–type TE dispersion. The CMC-Na acts as a stabilizer in the TE dispersion. Then, the dispersion was subjected to ultrasonic treatment for 40 min to allow it to fully disperse. After pouring the dispersion into a vacuum bottle, the filter paper was placed at the bottle mouth, and the pump was connected to the suction bottle for suction filtration. Finally, the N–type TE-modified paper was prepared after 7–8 h of filtration and heating the modified paper at 60 °C for 2 h. The P–type TE-modified paper was prepared with the same procedure. In this work, we prepared three different ratios of modified paper: 45%, 56%, and 67%, respectively.

### 2.3. Preparation of PTEGs and WPTEGs

The N–type- and P–type-modified papers were cut into strips with a size of 4 mm × 30 mm, which were served as TE legs. Then, these TE legs were alternately connected to Al foils in the order of “N–P–N–P”, forming a conductive path. After connecting the wires to both ends of the TE legs, a flexible PTEG was fabricated. On this basis, a superhydrophobic layer encapsulation is performed on a PTEG to obtain the WPTEG.

### 2.4. Characterization and Testing

The morphologies of the paper and thermoelectric materials were obtained by using a Supra 55 scanning electron microscope (Carl Zeiss Inc., Aalen, Germany). Energy Dispersive Spectrometer (EDS) elemental mappings and results were carried out by the JEM-ARM300F (JEOL Ltd., Tokyo, Japan). The mechanical flexibility of the device was conducted on an FT2000 flexible tester (Shanghai Mifang Electronic Technology Co., Ltd., Shanghai, China). The start angle was set to 0°, the end angle was set to 120°, the bending speed was set to 5°/s, and the bending cycle was 500 times. The voltage tests were conducted on the UT61E digital multimeter (UNI-T, Dongguan, China), and the current was measured by using the DMM 6500 (Keithley, Cleveland, OH, USA). The humidity test was conducted by using a humidity generator (FD-HG, Suzhou Furande Experimental Equipment Co., Ltd., Suzhou, China).

## 3. Results and Discussion

### 3.1. Preparation of PTEGs and WPTEGs

**Figure 2:**
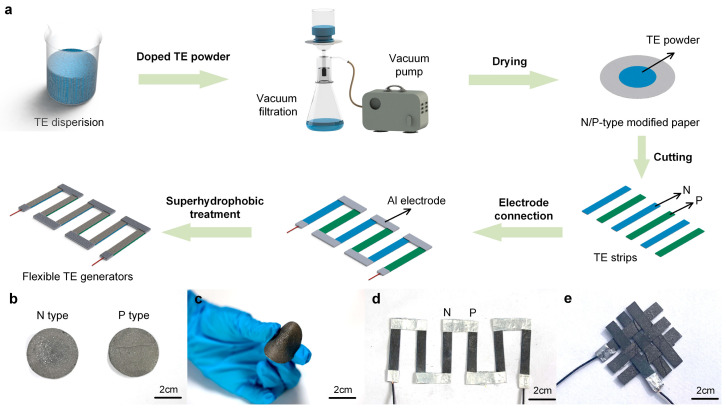
(**a**) Schematic diagram of the preparation process for a WPTEG. Digital photographs of (**b**) N–type- and P–type-modified cellulose papers after drying; (**c**) the modified paper with high flexibility; (**d**) a WPTEG with three units of N–P modules; (**e**) a WPTEG with woven structure.

Figure 2a illustrates a simple strategy for preparing the N–type- and P–type-modified cellulose papers and the PTEG. The TE dispersions of the N–type and P–type are prepared by dispersing each TE powder and carboxymethylcellulose sodium (CMC-Na) in deionized water. The N–type material is Bi_2_Te_3_ doped with Se, while the P–type material is Sb_2_Te_3_ doped with Bi. This is achieved by infiltrating TE materials into a cellulose paper matrix to fabricate N–type- and P–type-modified cellulose papers. By heating and drying at 60 °C for 2 h, the two modified papers are cut into the same strips (4 mm × 30 mm), serving as TE legs. Subsequently, these legs are alternately connected to the Al foil to form a conductive path. By now, the paper-based thermoelectric generator (PTEG) is successfully prepared. After the superhydrophobic layer encapsulation, the flexible WPTEG is finally fabricated. Figure 2b shows the pictures of N–type- and P–type-modified cellulose papers after drying. In addition, the modified papers have excellent flexibility and can get really close contact with curved surfaces, as shown in Figure 2c. The fabricated WPTEG is revealed in Figure 2d, which comprised three pairs of N–P modules. Moreover, due to the flexibility and tenacity of cellulose paper, a complex woven structure can be formed, as shown in Figure 2e.

### 3.2. Characterization of Modified Cellulose Papers

**Figure 3:**
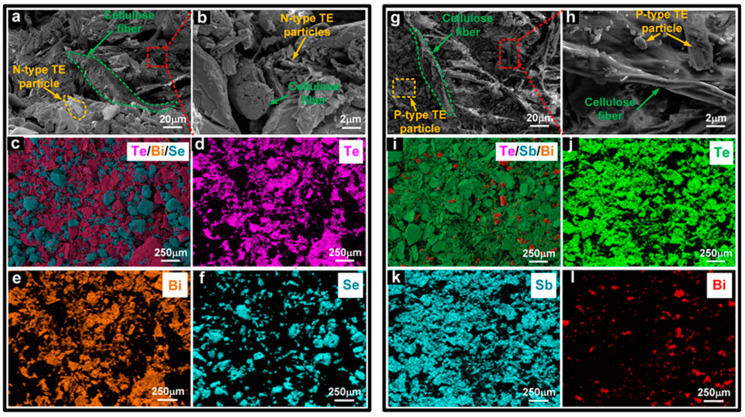
SEM images of (**a**) N–type-modified paper; (**b**) Enlarged view of the region enclosed by the red dashed rectangle in (a). (**c**–**f**) EDS elemental mappings and distributions of N–type-modified paper. SEM images of (**g**) P–type-modified paper; (**h**) an enlarged view of the paper. (**i**–**l**) EDS elemental mappings and distributions of P–type-modified paper.

Cellulose paper composed of adjacent and interlaced cellulose fibers exhibits lightweight, flexibility, hydrophily, and porosity and can be used for wearable devices. The surface morphology of cellulose paper is characterized by scanning electron microscopy (SEM), as shown in Figure S1. Figure 3a,b show the SEM images of N–type-modified paper. During the vacuum filtration, part of the N–type TE particles is deposited on the paper’s surface and adhered to the cellulose fibers, and the others penetrated deep into the gaps between fibers. The N–type TE particles adhered to the surface of the paper are adjacent to each other to form conductive pathways, and the particles in the fiber gaps serve as conductive supplements. As illustrated in Figure 3c–f, Energy Dispersive Spectrometer (EDS) elemental mappings are performed to further analyze the distributions of N–type TE particles on cellulose paper. Since the N–type material is Bi_2_Te_3_ doped with Se, the distributions of Te and Bi are highly overlapped, as shown in Figure 3d,e, while Se is distributed discretely in gaps of Bi_2_Te_3_. It could be seen that the N–type TE particles are spread roughly evenly across the cellulose paper. SEM images of P–type-modified paper are shown in Figure 3g,h. Similarly, P–type TE particles are deposited on the surface and gaps of cellulose fibers, forming the conductive pathways. Moreover, the EDS elemental mappings, as shown in Figure 3i–l, illustrate the overlapped Te and Sb, as well as relatively small amounts of Bi. The EDS results of N–type and P–type TE papers are depicted in Figure S2, implying that the atomic percentage of Bi:Te:Se is approximately 8.34:9.85:31.12 and Sb:Te:Bi is approximately 18.63:26.86:3.04.

**Figure 4:**
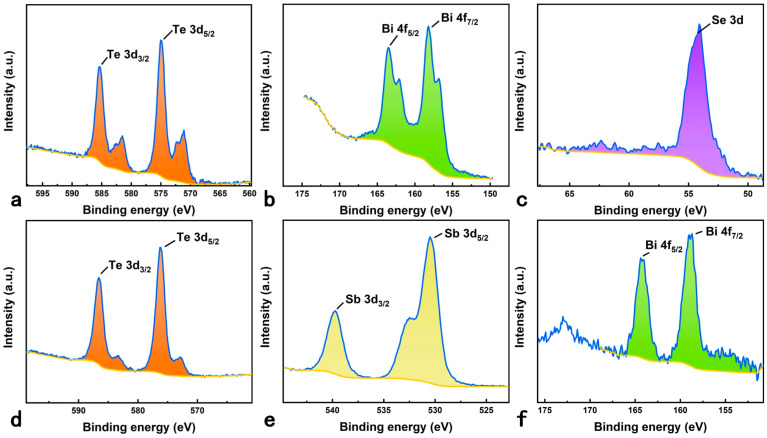
XPS spectra of (**a**) Te 3d, (**b**) Bi 4f, and (**c**) Se 3d regions of N–type-modified paper, respectively. XPS spectra of (**d**) Te 3d, (**e**) Sb 3d, and (**f**) Bi 4f regions of P–type-modified paper, respectively.

Figure 4a–c show the XPS peak-differentiation-imitating results for the Te 3d, Bi 4f, and Se 3d peaks of N–type-modified paper before superhydrophobic treatment. The binding energies for Te 3d_5/2_ and Te 3d_3/2_ are 575 eV and 585.3 eV, and the binding energies for Bi 4f_7/2_ and Bi 4f_5/2_ are 158.2 eV and 163.5 eV, respectively. The experimental values for the binding energies are close to the reported value [25,26]. The binding energy of Se 3d at 54.1 eV is consistent with the data reported in the literature [27]. Figure 4d–f show the XPS peak-differentiation-imitating results for the Te 3d, Sb 3d, and Bi 4f peaks in P–type-modified paper before superhydrophobic treatment. The binding energies for Te 3d_5/2_ and Te 3d_3/2_ are 576.3 eV and 586.6 eV, and the binding energies for Bi 4f_7/2_ and Bi 4f_5/2_ are 158.7 eV and 164.4 eV, respectively. The experimental values for the binding energies are close to the reported values [25,26]. The binding energies for Sb3d_5/2_ and Sb3d_3/2_ are 530.5 eV and 539.7 eV with a separation of 9.2 eV, and the position and separation of these two peaks are close to the reported value [28]. Figure S3 shows the XRD images of N–type and P–type-modified paper, respectively. The XRD peaks of the samples match well with the peaks of previously published single crystal samples [29,30,31]. These results confirmed the existence of the Te, Bi, and Se elements in N–type-modified paper, as well as the Te, Sb, and Bi elements in P–type-modified paper.

### 3.3. Performance of the PTEGs

**Figure 5:**
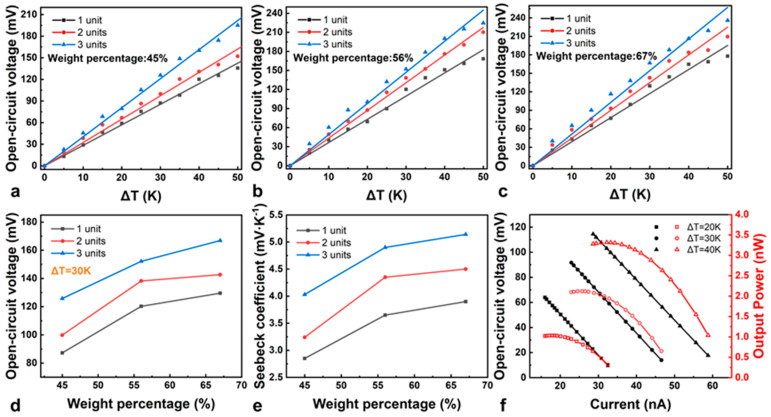
Open-circuit voltages of PTEGs prepared with (**a**) 45%, (**b**) 56%, and (**c**) 67% modified paper at temperature differences of 5 to 50 K. (**d**) Relationship between the open-circuit voltage of the PTEG and the weight percentage of the TE materials at a temperature difference of 30 K for the PTEGs with 1–3 units. (**e**) Relationship between the Seebeck coefficient of the PTEGs and the weight percentage of the TE materials for the PTEGs with 1–3 units. (**f**) Output voltage and output power curves.

In order to achieve a PTEG with a high voltage output, we investigated the weight percentages of TE materials in cellulose paper and the numbers of N–P modules, respectively. As shown in Figure 5a–c, for one unit of the N–P module, the open circuit voltage of PTEGs constantly increases with a rise in the weight percentages of TE materials (45% to 67%) and temperature differences (ΔT). When the weight percentages are the same, the open circuit voltage of PTEGs increases with the number of N–P modules. Thus, the performance of a PTEG is determined by the weight percentages of TE materials and the number of N–P modules, and the open circuit voltage of a PTEG reaches ~235.76 mV with a weight percentage of 67% and ΔT of 50 K. A TEG using similar materials can also achieve an output voltage of 200 mV [32]. We have attached a video showing a voltage above 200 mV. The open circuit voltages of PTEGs with different units and weight percentages at a ΔT of 30 K are extracted, as shown in Figure 5d. When the weight percentages are 45%, 56%, and 67%, PTEGs with three N–P modules generate open circuit voltages of 125.79 mV, 152.12 mV, and 166.83 mV, respectively. The large weight percentage allows more active materials to participate in power generation, thereby increasing the generated voltage. The Seebeck coefficient (*S*), which is defined as the change rate of the thermoelectric potential with temperature variation, is the key parameter that influences the thermoelectric performance. The *S* can be defined by Equation (1):

(1) $$S=\frac{dV}{dT}$$

where *V* is the open circuit voltage and *T* is the temperature. The *S* of PTEGs with different weight percentages and units can be calculated using the slope of a *dV* versus *dT* plot by linear fitting [33]. The Seebeck coefficients of the flexible PTEGs are summarized in Figure 5e. When the weight percentages of modified paper are 45%, 56%, and 67%, the *S* value is 4.03 mV·K^−1^, 4.9 mV·K^−1^, and 5.14 mV·K^−1^, respectively. In addition, we also tested the Seebeck coefficient values of individual N–type and P–type thermoelectric materials using a self-made heating and signal acquisition system, as shown in Figure S4. As shown in Figure S5, the maximum Seebeck coefficient for a single N–type TE leg and a single P–type TE are −789 μV·K^−1^ and 798 μV·K^−1^, respectively. In order to observe the thickness of the deposited thermoelectric materials, we measured the height of the different content of thermoelectric materials deposited on cellulose paper using a step gauge. The thickness variation of the TE materials with different weight percentages is shown in Figure S6. The thickness of the deposited layer of modified paper also increases with the weight of the thermoelectric materials. As shown in Figure 5d and Figure S12, as the thickness of the thermoelectric materials increases, more active materials participate in power generation, resulting in a higher voltage output and an increase in the Seebeck coefficient. Figure S7 shows SEM images of the cross-section of N–type- and P–type-modified cellulose paper. We use a microtome to cut the modified paper to observe the cross-section, and the cutting process causes the modified paper to deform and thin. However, it can still be observed from the cross-sectional SEM images that the mass percentage of the TE material increases, and the thickness of the modified paper also increases. As it turns out, the obtained *S* value is impressive by comparing with the previous reports [34]. Figure 5f shows the variations of the external circuit voltage and output power of a PTEG with three modules as the current changes. The value of output power is the product of an external circuit voltage and current, and can be defined by Equation (2):

(2) $$P={\left(\frac{U_{0}}{R_{i}+R_{l}}\right)}^{2}\cdot R_{l}$$

where *U*_0_ is the open-circuit voltage of the TE device, *R_i_* is the internal resistance of the PTEG, and *R_l_* is the load resistance of the circuit. The current in the circuit decreases and the voltage of the resistance increases as the load increases. The maximum output power is 1.03 nW, 2.12 nW, and 3.32 nW, when the ΔT is 20 K, 30 K, and 40 K, respectively. Also, the TE performance of a PTEG is much higher than that of previously reported flexible TEGs [35,36,37,38,39,40,41] (Figure S8). Therefore, the PTEG at the nanowatt level is a promising candidate power supply device to be applied to low-power wearable chips in the future [42].

The prepared PTEG can be applied to human skin for power generation by utilizing the temperature difference between the ambient air and the body. As shown in Figure S9a, the prepared PTEG attached to the human arm can generate an open-circuit voltage of ~13.85 mV. When encountering rainy days or sweating, the performance of PTEGs is badly affected due to the super water absorption of cellulose paper, as shown in Figure S9b.

### 3.4. Characterization and Performance of the WPTEGs

**Figure 6:**
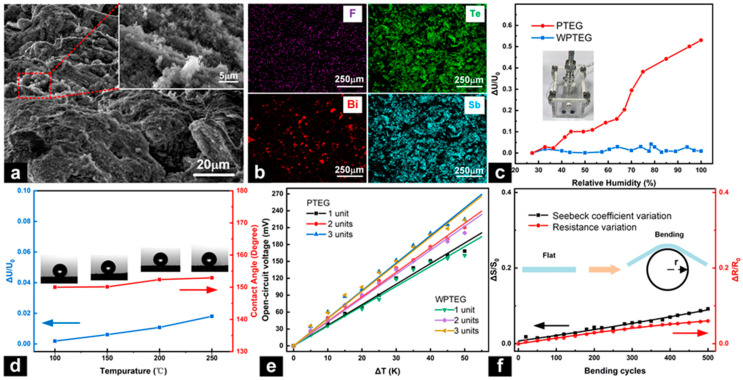
(**a**) SEM image of the P–type-modified paper after superhydrophobic treatment. The insert shows an enlargement of the superhydrophobic coating in the region enclosed by the red dashed rectangle. (**b**) EDS maps of the superhydrophobic P–type-modified paper. (**c**) Voltage variation rates of the PTEG and WPTEG with the relative humidity. (**d**) Voltage change rates and contact angles at different baking temperatures. (**e**) Open-circuit voltages of the PTEG and WPTEG with different numbers of units at different ΔT. (**f**) Mechanical stability of the WPTEG.

Figure 6a shows the SEM images of P–type-modified paper after superhydrophobic treatment. The inset suggests that the superhydrophobic coating is compounded from micrometer-size clusters and aggregates. Figure 6b shows the EDS diagrams of superhydrophobic P–type-modified paper. Aside from the Te, Bi, and Sb elements, a small amount of F element is detected. The content of each element is shown in Figure S10. It is widely known that cellulose paper is superhydrophilic and has a contact angle of <5° (Figure S11). The wettability of N– and P–type-modified papers is similar to that of cellulose paper, as shown in Figure S12a. Meanwhile, the contact angle of the modified paper turns into ~152.4° after superhydrophobic treatment, indicating the water resistance of the modified paper. Figure S13 shows that the modified paper, before superhydrophobic treatment, rapidly absorbs water and curls up in contact with the solution and reaches saturation, while the modified paper, after superhydrophobic treatment, isolated the water from the device and maintains the morphology and stiffness after 10 min, reflecting the waterproof properties and adaptability to humidity or water. Figure S14 shows that the WPTEG has good flexibility. In Figure S12b, the XPS peak-differentiation-imitating result for F 1s on superhydrophobic P–type-modified paper also demonstrated the presence of fluorine groups. To verify the water resistance, the voltage variation rates of the PTEG and WPTEG in different humidities are measured, as illustrated in Figure 6c. With the increase in humidity, the voltage variation of the PTEG is very obvious. When the relative humidity was close to 100%, the voltage change rate reaches over 50% at ΔT of 20 K, implying that the PTEG is highly affected by humidity. Instead, the WPTEG exhibited a relatively stable voltage output with a change rate of less than 5%, illustrating its excellent water-resisting property. The inset of Figure 6c shows the humidity chamber. Also, after 50 wet–dry cycles, the changes in the Seebeck coefficient and internal resistance are less than 5% and 4% when the humidity reached ~100%, as shown in Figure S15. The slight changes in the internal resistance and Seebeck coefficient indicate that the modified paper has high stability, waterproofing, and durability.

To illustrate the stability of the device, the WPTEG is baked in an oven for 30 min at different temperatures (100 °C, 150 °C, 200 °C, 250 °C). With the increase in the baking temperature, the voltage variation rate remained basically stable (within 2%), as depicted in Figure 6d. Meanwhile, the contact angle still maintains over 150°, resulting in excellent high-temperature resistance (red line in Figure 6d). Figure 6e shows the open-circuit voltage of the PTEG and WPTEG with different units at different ΔT. The results illustrate that the superhydrophobic coating rarely affects the performance of the WPTEG. Additionally, the mechanical stability of the WPTEG is explored. As shown in Figure 6f, after 500 bending cycles, the changes in the Seebeck coefficient and internal resistance are less than 10% and 6% with a bending angle of ~120°. This small change in the internal resistance and Seebeck coefficient indicate that the modified paper has good stability and durability, and has promising application prospects in various heat source surfaces. Subsequently, the output voltages of the PTEG and WPTEG attached to dry and wet arms are acquired. As shown in Figure S12c, the output voltage of the PTEG on a dry arm is 13.85 mV (PTEG-1), while the voltage of the PTEG on a wet arm is 2.27 mV (PTEG-2). This means that PTEGs are unable to maintain a stable and good power supply capability under wet conditions. For the WPTEG on a dry arm, an output voltage of 12.23 mV is achieved (WPTEG-1). When the WPTEG is on a wet arm, the output voltage is 11.85 mV (WPTEG-2). The output voltage of the WPTEG on a wet arm is slightly smaller than that on a dry arm since a small amount of water seeped into the paper. Nevertheless, the performance of the WPTEG is demonstrated to be excellent. The results from what have been discussed above suggest that the WPTEG has potential applications in energy collection on the human body, particularly on sweat or humid skin.

### 3.5. Applications of the WPTEGs

**Figure 7:**
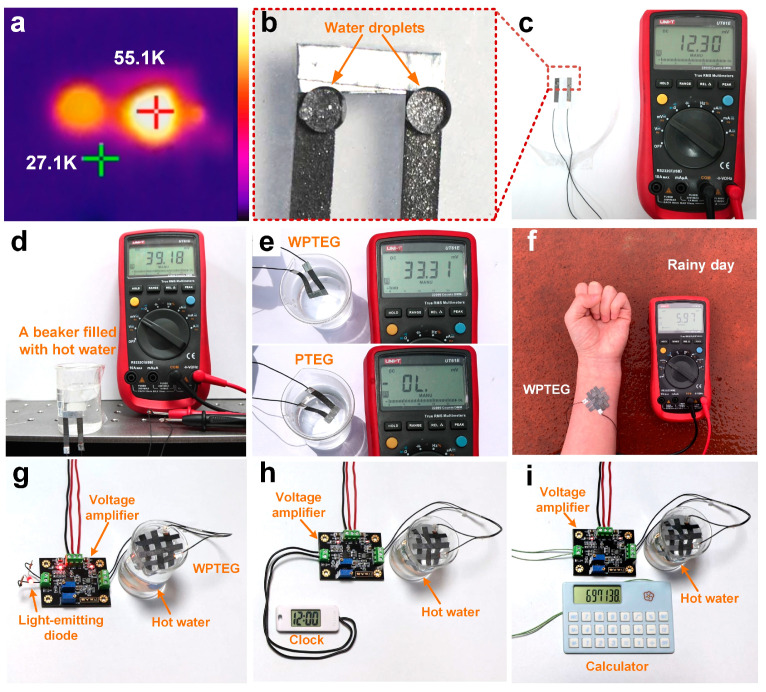
(**a**–**c**) Power generation from water droplets. (**a**) Infrared image of two water droplets on the WPTEG. (**b**) Photograph of two water droplets on the WPTEG. The temperatures of the two droplets were 27.1 and 55.1 K. (**c**) Output voltage of the two water droplets on the WPTEG. (**d**) Power generation from a beaker filled with hot water. (**e**) Comparison of the output voltages of the PTEG and WPTEG in hot water. (**f**) Woven WPTEG attached to an arm on a rainy day. Photographs of the WPTEG powering (**g**) a diode, (**h**) a clock, and (**i**) a calculator by harvesting energy from hot water.

The WPTEGs can not only convert human heat into electrical energy, but also collect waste heat from hygrothermal or water environments. As shown in Figure 7c, two hot water droplets placed on one side of the WPTEG generate an output voltage of 12.3 mV. Moreover, droplets always keep a spherical shape due to the water resistance of the WPTEG, as depicted in Figure 7b. Figure 7a shows the infrared image of the two droplets with a maximum temperature difference of ~28 K between the droplet and air. We drip the left droplet and the right droplet in sequence, resulting in a lower temperature of the left droplet compared with the right one. Figure 7d illustrates a WPTEG pasted onto the surface of a beaker filled with hot water, and an output voltage of 39.18 mV is achieved. It indicated that the WPTEG has excellent mechanical flexibility and is suitable for the energy collection of various complex curved heat sources. Figure 7e shows the WPTEG and PTEG are immersed in water at ~100 °C, respectively. The output voltage of the WPTEG is 33.31 mV, while that of the PTEG is 0 mV. Many devices lose performance in an underwater environment. To verify that the WPTEG can harvest thermal energy from underwater environments, we simulate a scenario where thermal energy is harvested in an underwater environment. As shown in Figure S16a, we stick the WPTEG to our wrist and insert it into cold water (25 degrees, 40 s). The output voltage of the WPTEG is 4.94 mV. Also, we stick the WPTEG onto a hose filled with hot water (100 °C) and immerse the hose deep into cold water. The output voltage of the WPTEG is 10.33 mV, as shown in Figure S16b. The results show that the WPTEG is still able to harvest thermal energy from the human body, water pipes, and other heat sources in underwater environments. Prior to application, the interface is tightly encapsulated so that the WPTEG is not affected by short circuits in the water. Accordingly, the WPTEG as a wearable power source has huge potential in practical applications, such as underwater use. Furthermore, a woven WPTEG can be obtained owing to the pliability of the paper. Figure 7f shows an intersectant woven structure with three N–P modules that are set on the arm on a rainy day. Meanwhile, an output voltage of 5.97 mV is obtained, illustrating the applicability of the WPTEG in various weather environments.

In recent years, flexible generators have been considered a promising power source for wearable electronic devices, overcoming traditional batteries’ shortcomings, such as frequent charging and environmental pollution. A reliable method is to use a power management circuit to regulate the voltage, and then directly integrate the generator with functional electronic devices to form a self-powered microsystem. In this work, the fabricated WPTEG is used to power portable electronic devices (diodes, clocks, and calculators) to verify their feasibility as wearable power sources (Figure 7g–i). Due to its output performance at the millivolt level, a PTEG cannot directly drive ordinary electronic devices. Therefore, we use a power management circuit to boost the output voltage. In practical applications, the WPTEG is placed in hot water as the power source, and the output voltage from the WPTEG is amplified to light up the diode and power the clock and calculator, illustrating the reliability and availability of the power generation of the WPTEG in an underwater environment.

## 4. Conclusions

In summary, a waterproof paper-based wearable thermoelectric generator (WPTEG) for collecting low-grade thermal energy from the human body and serving as a power supply for portable devices has been demonstrated. The paper-based generator is composed of TE-modified paper which was prepared by using a vacuum filtration process and Al electrode connection. Such a device has a high Seebeck coefficient of 5.14 mV·K^−1^, and a WPTEG with three units of N–P modules could obtain a maximum output power of ~3.32 nW at a ΔT of 40 K. After the superhydrophobic layer encapsulation, the formed WPTEG can be used in high humidity and underwater environments, and the performance is not infected basically. Moreover, the WPTEG presents excellent high-temperature resistance and good stability. Eventually, the device can provide stable power for various portable electronics in water environments, revealing huge potential in practical applications, such as underwater use.

## Data Availability

Data is unavailable due to privacy.

## Supplementary Materials

The following supporting information can be downloaded at: https://www.mdpi.com/article/10.3390/ma17102338/s1. Figure S1: SEM images of cellulose paper; Figure S2: (a) Energy dispersive spectroscopy results and composition element content of N-type Materials. (b) Energy dispersive spectroscopy results and composition element content of P-type Materials; Figure S3: (a) XRD pattern of N-type modified paper before superhydrophobic treatment. (b) XRD pattern of P-type modified paper before superhydrophobic treatment; Figure S4: A self-made heating and signal acquisition system; Figure S5: The Seebeck coefficients for individual (a) N-type and (b) P-type thermoelectric materials with different the weight percentages of TE materials (45% to 67%) and temperature differences (ΔT); Figure S6: Changes in the thickness of modified paper film; Figure S7: SEM images of the thickness of the deposited layer of (a–c) N-type and (d–f) P-type modified cellulose paper increasing with the weight (45% to 67%) of the thermoelectric material; Figure S8: Comparison of the open-circuit voltage and Seebeck coefficient of previously reported flexible TEGs and our PTEG; Figure S9: Photographs of human skin generating electricity with the PTEGs from (a) dry arm; (b) wet arm; Figure S10: Energy Dispersion Spectroscopy results and composition element content of waterproof P-type modified paper; Figure S11: Contact angle of cellulose paper; Figure S12: (a) Comparison of the contact angles of the modified paper before and after superhydrophobic treatment. (b) XPS peak results of F 1s for the superhydrophobic P-type modified paper. (c) Output voltages of the PTEG and WPTEG attached to a dry arm and a wet arm; Figure S13: Optical images of modified paper (a) before and (b) after superhydrophobic treatment submerged in water; Figure S14: Twisted WPTEG. (b) Curved WPTEG; Figure S15: The material durability of WPTEG after 50 cycles; Figure S16: (a) Photograph of a WPTEG absorbing body heat from an underwater environment (25 degree, 40 s). (b) Photograph of a WPTEG absorbing thermal power from a hose filled with hot water (100 °C) in an underwater environment.

## References

[1] Fu Y., Cheng Y., Wei Q., Zhao Y., Zhang W., Yang Y., Li D. (2022). Multifunctional Biomass Composite Aerogel Co-modified by MXene and Ag Nanowires for Health Monitoring and Synergistic Antibacterial Applications. Appl. Surf. Sci..

[2] Martinez-Hernandez U., Firouzy S., Mehryar P., Meng L., Childs C., Buis A., Dehghani-Sanij A.A. (2023). Human-in-the-loop Layered Architecture for Control of A Wearable Ankle–foot Robot. Robot. Auton. Syst..

[3] Tan P., Han X., Zou Y., Qu X., Xue J., Li T., Wang Y., Luo R., Cui X., Xi Y. (2022). Self-Powered Gesture Recognition Wristband Enabled by Machine Learning for Full Keyboard and Multicommand Input. Adv. Mater..

[4] Sarkar A., Shrotriya P., Chandra A., Hu C. (2019). Chemo-economic Analysis of Battery Aging and Capacity Fade in Lithium-ion Battery. J. Energy Storage.

[5] Klink J., Hebenbrock A., Grabow J., Orazov N., Nylén U., Benger R., Beck H.-P. (2022). Comparison of Model-Based and Sensor-Based Detection of Thermal Runaway in Li-Ion Battery Modules for Automotive Application. Batteries.

[6] Ren Z., Zheng Q., Wang H., Guo H., Miao L., Wan J., Xu C., Cheng S., Zhang H. (2020). Wearable and Self-cleaning Hybrid Energy Harvesting System Based on Micro/nanostructured Haze Film. Nano Energy.

[7] Tang Y., Lei Y., Li H., Wu Y., Li Y., Liu S., Wang H., Liu X. (2023). An Effective Oxygen Vacancy Restrain Method for Flexible Perovskite Solar Cells with Enhanced Performance and Bending Resistance. Appl. Surf. Sci..

[8] Li J., Zhao C., Xia K., Liu X., Li D., Han J. (2019). Enhanced Piezoelectric Output of the PVDF-TrFE/ZnO Flexible Piezoelectric Nanogenerator by Surface Modification. Appl. Surf. Sci..

[9] Zhao Z., Dai Y., Dou S.X., Liang J. (2021). Flexible Nanogenerators for Wearable Electronic Applications Based on Piezoelectric Materials. Mater. Today Energy.

[10] Jeon Y.P., Park J.H., Kim T.W. (2019). Highly flexible Triboelectric Nanogenerators Fabricated Utilizing Active Layers with A ZnO Nanostructure on Polyethylene Naphthalate Substrates. Appl. Surf. Sci..

[11] Yun J., Jayababu N., Kim D. (2020). Self-powered Transparent and Flexible Touchpad Based on Triboelectricity Towards Artificial Intelligence. Nano Energy.

[12] Lu Y., Li X., Cai K., Gao M., Zhao W., He J., Wei P. (2021). Enhanced-Performance PEDOT:PSS/Cu_2_Se-Based Composite Films for Wearable Thermoelectric Power Generators. ACS Appl. Mater. Interfaces.

[13] Wang Y., Yang L., Shi X.L., Shi X., Chen L., Dargusch M.S., Zou J., Chen Z.G. (2019). Flexible Thermoelectric Materials and Generators: Challenges and Innovations. Adv. Mater..

[14] Pandiyarasan V., Suhasini S., Archana J., Navaneethan M., Majumdar A., Hayakawa Y., Ikeda H. (2017). Fabrication of Hierarchical ZnO Nanostructures on Cotton Fabric for Wearable Device Applications. Appl. Surf. Sci..

[15] Du Y., Chen J., Meng Q., Xu J., Paul B., Eklund P. (2020). Flexible Ternary Carbon Black/Bi_2_Te_3_ based Alloy/polylactic Acid Thermoelectric Composites Fabricated by Additive Manufacturing. J. Mater..

[16] Shi T., Chen M., Zhang C., Mao Z., Liang J., Liu Z., Zhang J., Zhang Q., Pan L., Wang Y. (2023). Modifying Carbon Fiber Fabric for Flexible Thermoelectric Energy Conversion. Appl. Surf. Sci..

[17] Lin Y., Liu J., Wang X., Xu J., Liu P., Nie G., Liu C., Jiang F. (2019). An Integral P-n Connected All-graphene Fiber Boosting Wearable Thermoelectric Energy Harvesting. Compos. Commun..

[18] Zhang Y., Dai Y., Xia F., Zhang X. (2022). Gelatin/polyacrylamide Ionic Conductive Hydrogel with Skin Temperature-triggered Adhesion for Human Motion Sensing and Body Heat Harvesting. Nano Energy.

[19] Li H., Zong Y., Ding Q., Han W., Li X. (2021). Paper-based Thermoelectric Generator Based on Multi-walled Carbon Nanotube/carboxylated Nanocellulose. J. Power Sources.

[20] Kim S., Mo J.-H., Jang K.-S. (2019). Solution-Processed Carbon Nanotube Buckypapers for Foldable Thermoelectric Generators. ACS Appl. Mater. Interfaces.

[21] Zhan Z., Lin R., Tran V.-T., An J., Wei Y., Du H., Tran T., Lu W. (2017). Paper/Carbon Nanotube-Based Wearable Pressure Sensor for Physiological Signal Acquisition and Soft Robotic Skin. ACS Appl. Mater. Interfaces.

[22] Chen S., Song Y., Xu F. (2018). Flexible and Highly Sensitive Resistive Pressure Sensor Based on Carbonized Crepe Paper with Corrugated Structure. ACS Appl. Mater. Interfaces.

[23] Karan S.K., Maiti S., Lee J.H., Mishra Y.K., Khatua B.B., Kim J.K. (2020). Recent Advances in Self-powered Tribo-/piezoelectric Energy Harvesters: All-in-one Package for Puture Smart Technologies. Adv. Funct. Mater..

[24] Sun T., Zhou B., Zheng Q., Wang L., Jiang W. (2020). Stretchable fabric generates electric power from woven thermoelectric fibers. Nat. Commun..

[25] Kumar R., Bhatt R., Tewary A., Debnath A., Bhatt P., Mani N., Jha P., Patro P., Bhattacharya S., Pathak M. (2022). Synergistic Effect of Zn Doping on Thermoelectric Properties to Realize a High Figure-of-merit and Conversion Efficiency in Bi_2−x_Zn_x_Te_3_ Based Thermoelectric Generators. J. Mater. Chem. C.

[26] Bae E.J., Kang Y.H., Jang K.-S., Lee C., Cho S.Y. (2016). Solution Synthesis of Telluride-based Nano-barbell Structures Coated with PEDOT: PSS for Spray-printed Thermoelectric Generators. Nanoscale.

[27] Jin R., Chen G., Pei J., Sun J., Wang Y. (2011). Controllable Synthesis and Electrochemical Hydrogen Storage Properties of Sb_2_Se_3_ Ultralong Nanobelts with Urchin-like Structures. Nanoscale.

[28] Zhang C., Li Z., Guo Y., Niu X., Liang X., Zhou D., Zhu H., Chen J., Mai Y. (2017). Controllable Synthesis and Properties of Sb_2_Se_3_ Nanorods Synthesized by Hot-injection Method. J. Nanosci. Nanotechnol..

[29] Yuan Z., Tang X., Xu Z., Li J., Chen W., Liu K., Liu Y., Zhang Z. (2018). Screen-printed Radial Structure Micro Radioisotope Thermoelectric Generator. Appl. Energ..

[30] Francioso L., De Pascali C., Farella I., Martucci C., Cretì P., Siciliano P., Perrone A. (2011). Flexible Thermoelectric Generator for Ambient Assisted Living Wearable Biometric Sensors. J. Power Sources.

[31] Kim S.J., Choi H., Kim Y., We J.H., Shin J.S., Lee H.E., Oh M.-W., Lee K.J., Cho B.J. (2017). Post Ionized Defect Engineering of The Screen-printed Bi_2_Te_2.7_Se_0.3_ Thick Film for High Performance Flexible Thermoelectric Generator. Nano Energy.

[32] Rojas J.P., Conchouso D., Arevalo A., Singh D., Foulds I.G. (2017). Paper-based origami flexible and foldable thermoelectric nanogenerator. Nano Energy.

[33] Wang Y., Zhang S., Deng Y. (2016). Flexible Low-grade Energy Utilization Devices Based on High-performance Thermoelectric Polyaniline/tellurium Nanorod Hybrid Films. J. Mater. Chem. A.

[34] Mulla R., Jones D.R., Dunnill C.W. (2020). Thermoelectric Paper: Graphite Pencil Traces on Paper to Fabricate a Thermoelectric Generator. Adv. Mater. Technol..

[35] Bae E.J., Kang Y.H., Lee C. (2017). Engineered nanocarbon mixing for enhancing the thermoelectric properties of a telluride-PEDOT: PSS nanocomposite. J. Mater. Chem. A.

[36] Liang L., Wang M., Wang X., Peng P., Liu Z. (2022). Initiating a stretchable, compressible, and wearable thermoelectric generator by a spiral architecture with ternary nanocomposites for efficient heat harvesting. Adv. Funct. Mater..

[37] Li Y., Lou Q., Yang J., Cai K., Liu Y., Lu Y. (2022). Exceptionally high power factor Ag_2_Se/Se/polypyrrole composite films for flexible thermoelectric generators. Adv. Funct. Mater..

[38] Wang Y., Hong M., Liu W.D., Shi X.L., Xu S.D. (2020). Bi_0. 5_Sb_1. 5_Te_3_/PEDOT: PSS-based flexible thermoelectric film and device. Chem. Eng. J..

[39] Lu Y., Qiu Y., Cai K., Li X., Gao M., Jiang C. (2020). Ultrahigh performance PEDOT/Ag_2_Se/CuAgSe composite film for wearable thermoelectric power generators. Mater. Today Phys..

[40] Wang Y., Pang H., Guo Q., Tsujii N., Baba T., Baba T. (2020). Flexible n-Type abundant chalcopyrite/PEDOT: PSS/graphene hybrid film for thermoelectric device utilizing low-grade heat. ACS Appl. Mater. Interfaces.

[41] Mallick M.M., Rösch A.G., Franke L., Ahmed S., Gall A., Geßwein H. (2020). High-performance Ag-Se-based n-type printed thermoelectric materials for high power density folded generators. ACS Appl. Mater. Interfaces.

[42] Zheng C., Xiang L., Jin W., Shen H., Zhao W., Zhang F., Di C.A., Zhu D. (2019). A Flexible Self-powered Sensing Element with Integrated Organic Thermoelectric Generator. Adv. Mater. Technol..

