# Supplementary material for: A Waterproof Flexible Paper-Based Thermoelectric Generator for Humidity and Underwater Environments
Source: Materials (Basel). 2024 May 14;17(10):2338. doi: 10.3390/ma17102338 (PMC11123197; doi:10.3390/ma17102338)
Supplement: Supplementary file 1 [file materials-17-02338-s001.zip › materials-2982276-supplementary/materials-2982276-SM/materials-2982276-supplementary.pdf]

Supporting Information

A Waterproof Flexible Paper-Based Thermoelectric Generator for Humidity and Underwater Environments

Yiduo Huang [1], Wenfeng Wang [1], Sijia Chang [1], Aida Bao [1], Yuan Liu [2,*], Ruirui Li [1,*] and Jijun Xiong [1]

[1] State Key Laboratory of Dynamic Measurement Technology, North University of China, Taiyuan 030051, China; huangyiduoz@163.com (Y.H.); 15383466221@163.com (W.W.); csj15035900484@163.com (S.C.); baoaida@126.com (A.B.); xiongjijun@nuc.edu.cn (J.X.)

[2] Shenzhen Institute of Advanced Technology, Chinese Academy of Sciences, 1068 Xueyuan Avenue, Shenzhen 518055, China

* Correspondence: y.liu1@siat.ac.cn (Y.L.); liruirui@nuc.edu.cn (R.L.)

The surface morphology of the original cellulose paper is characterized by scanning electron microscopy (SEM). Figure S1 shows the original paper at different scale bars. Cellulose paper composed of adjacent and interlaced cellulose fibers exhibits lightweight, flexibility, hydrophily, and porosity and can be used for wearable devices.

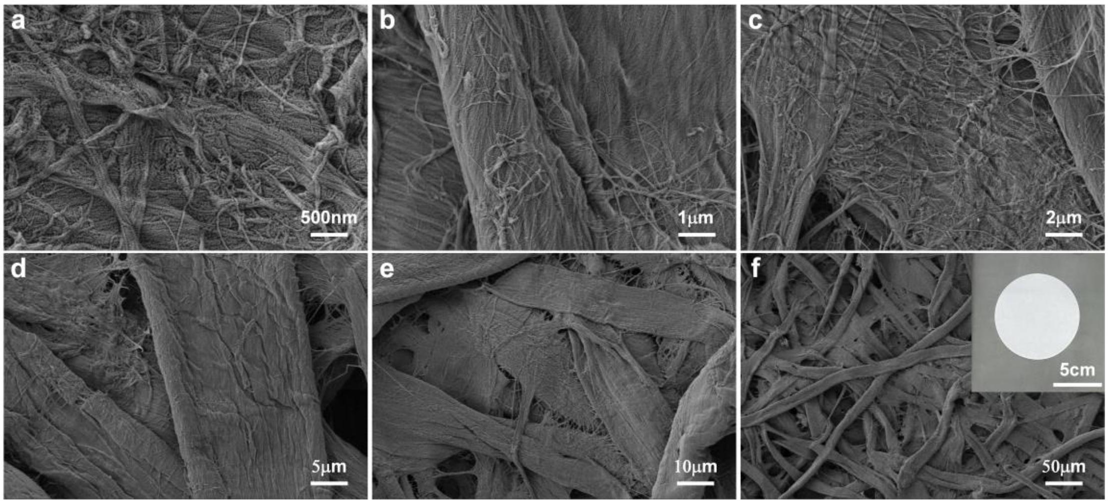

Figure S1. SEM images of cellulose paper.

By analyzing N-type modified paper and P-type modified paper, the elemental composition and content can be obtained. N-type modified paper is composed of C, O, Se, Te, Bi, and the atomic percentages indicate that Se:Te:Bi is approximately 31.1:9.9:8.3. P-type modified paper is composed of C, O, Sb, Te, Bi, and the atomic percentages indicate that Sb:Te:Bi is approximately 18.6:26.9:3. The C and O element are from the cellulose paper.

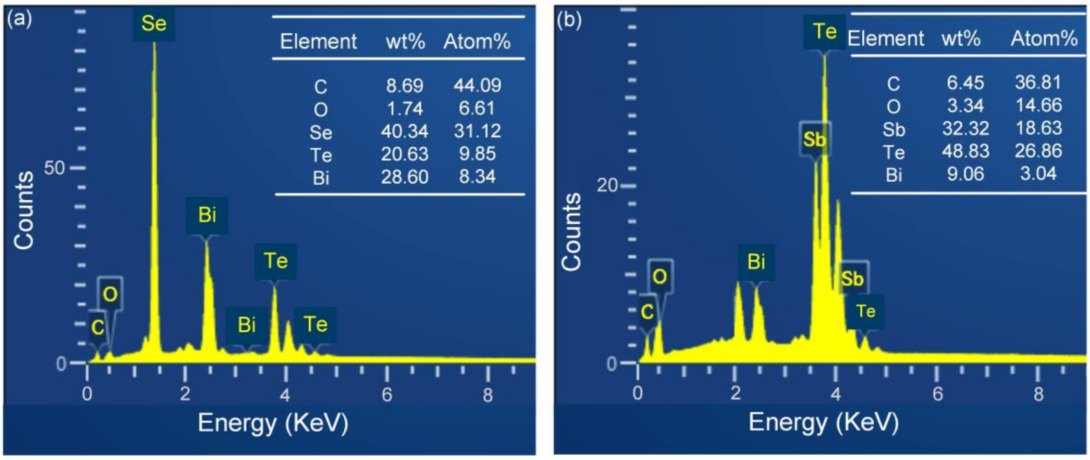

Figure S2. (a) Energy dispersive spectroscopy results and composition element content of N-type Materials. (b) Energy dispersive spectroscopy results and composition element content of P-type Materials.

Figures S3 shows the XRD images of N-type and P-type modified paper before superhydrophobic treatment, respectively. The XRD peaks of the samples match well with the peaks of previously published single crystal samples [1–3].

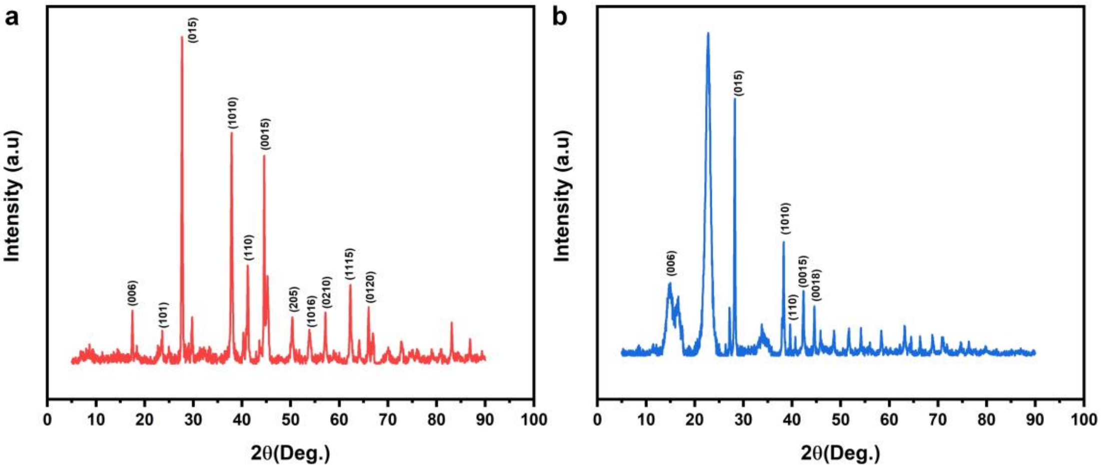

Figure S3. (a) XRD pattern of N-type modified paper before superhydrophobic treatment. (b) XRD pattern of P-type modified paper before superhydrophobic treatment.

Figure S4 shows that Seebeck coefficient was measured using a self-made heating and signal acquisition system that establishes a stable temperature difference on the material surface.

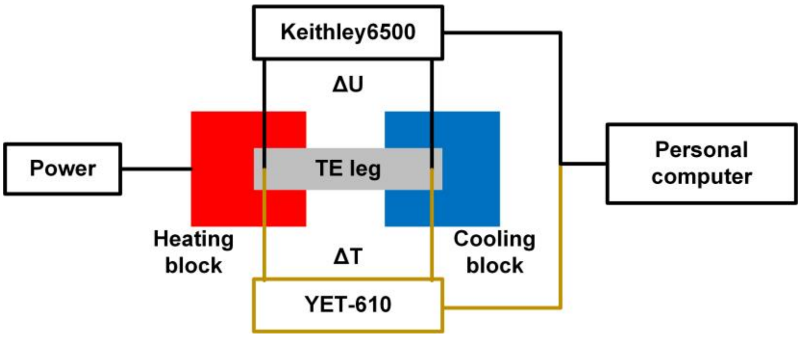

Figure S4. A self-made heating and signal acquisition system.

As shown in Figure S5, the maximum Seebeck coefficient for a single N-type TE leg and a single P-type TE are-789μV·K^{-1} and 798μV·K^{-1}, respectively.

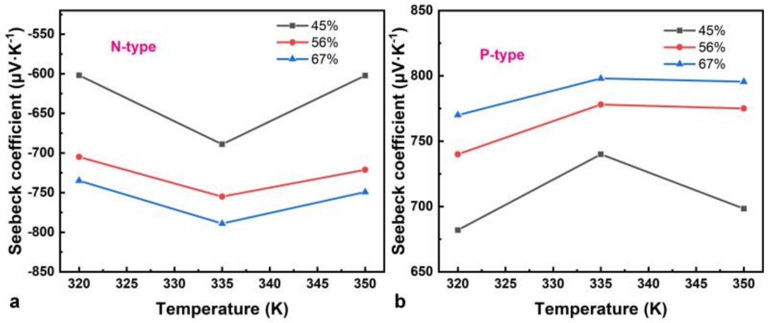

Figure S5. The Seebeck coefficients for individual (a) N-type and (b) P-type thermoelectric materials with different the weight percentages of TE materials (45% to 67%) and temperature differences (ΔT).

In order to observe the thickness of deposited thermoelectric materials, we measured the height of different content of thermoelectric materials deposited on cellulose paper using a step gauge. The thickness variation of TE materials with different weight percentages is shown in Figure S6. The thickness of the deposited layer of modified paper also increases with the weight of the thermoelectric materials.

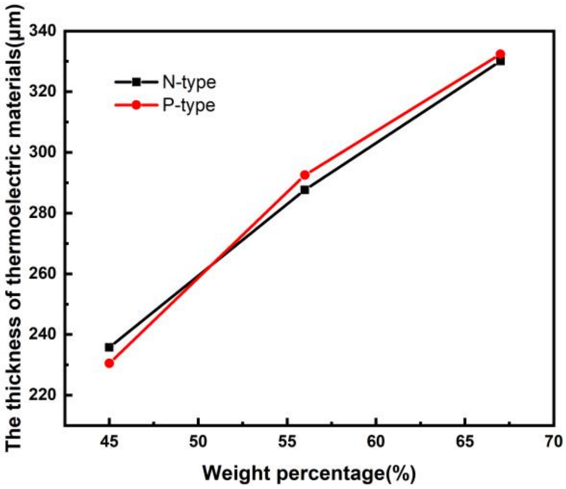

Figure S6. Changes in the thickness of modified paper film.

Figure S7 shows SEM images of the cross-section of N-type and P-type modified cellulose paper.

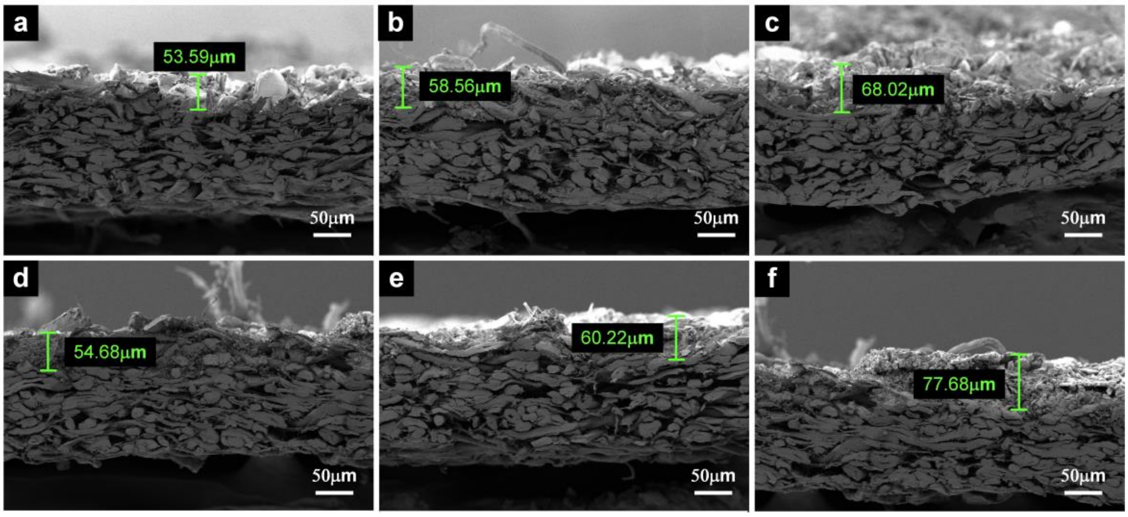

Figure S7. SEM images of the thickness of the deposited layer of (a-c) N-type and (d-f) P-type modified cellulose paper increasing with the weight (45% to 67%) of the thermoelectric material.

Figure S8 shows a comparative analysis with similar organic-inorganic composite paper-based thermoelectric materials can be added in the paper. The TE performance of PTEG is much higher than that of previously reported flexible TEGs.

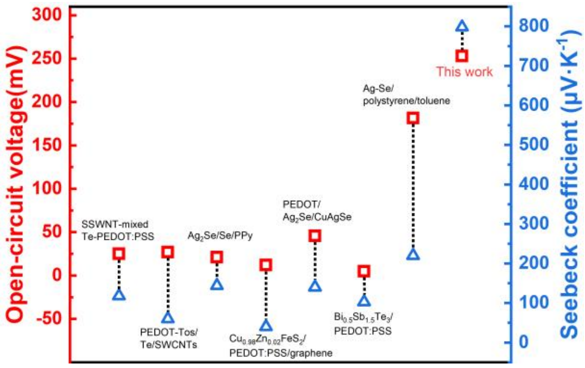

Figure S8. Comparison of the open-circuit voltage and Seebeck coefficient of previously reported flexible TEGs and our PTEG.

Figure S9 shows the output voltage of PTEG attached to the dry and wet arms. The former PTEG can generate an open-circuit voltage of ~13.85 mV, while the performance of PTEG on wet arm was badly affected due to the high water-absorbing quality of cellulose paper, and an open-circuit voltage of ~2.27 mV can only be generated.

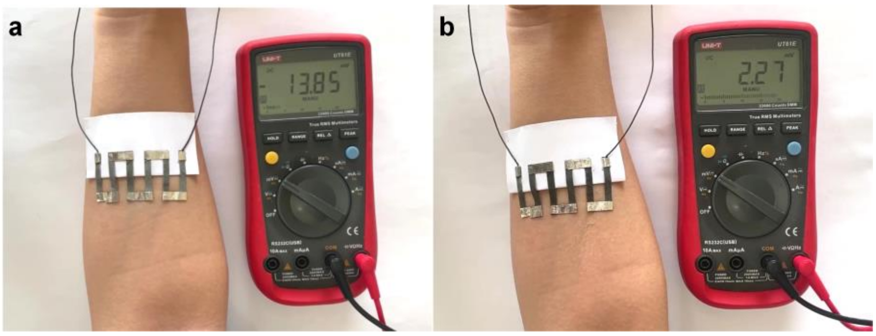

Figure S9. Photographs of human skin generating electricity with the PTEGs from (a) dry arm; (b) wet arm.

By analyzing the P-type modified paper with superhydrophobic layer encapsulation, the elemental composition and content can be obtained. A small number of F ele-

ment is detected, indicating the introduction of fluorine group in the modified paper. The P-type modified paper with superhydrophobic layer encapsulation is composed mainly of F, Sb, Te and Bi.

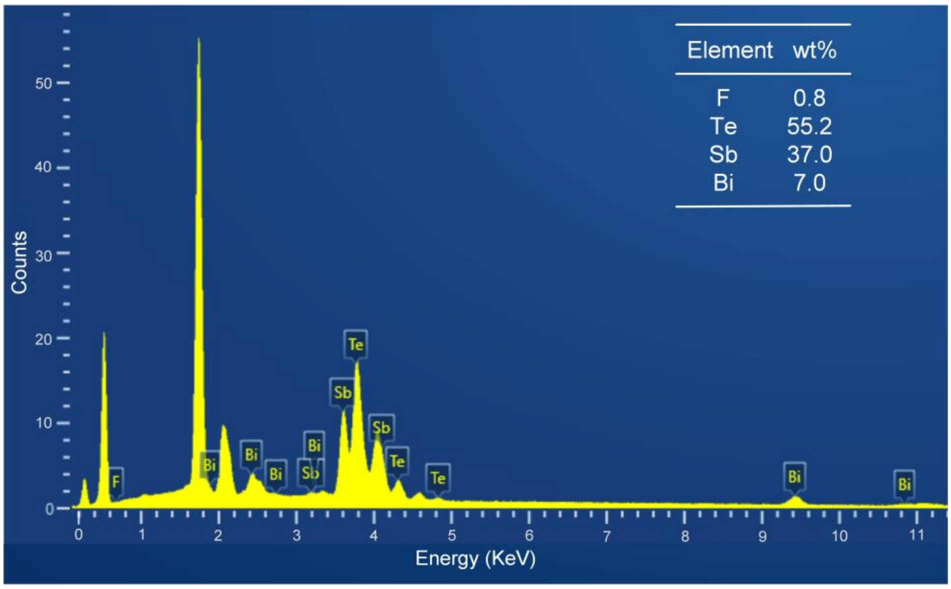

Figure S10. Energy Dispersion Spectroscopy results and composition element content of waterproof P-type modified paper.

Figure S11 shows that the contact angle of cellulose paper is ~1.5°, indicating the superhydrophilicity of cellulose paper.

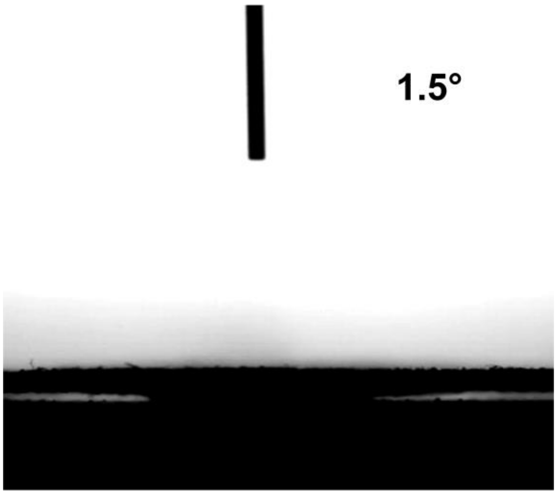

Figure S11. Contact angle of cellulose paper.

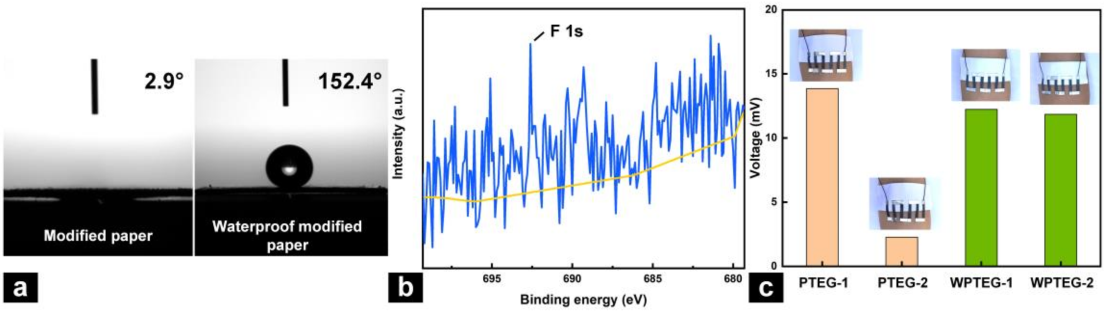

Figure S12. (a) Comparison of the contact angles of the modified paper before and after superhydrophobic treatment. (b) XPS peak results of F 1s for the superhydrophobic P-type modified paper. (c) Output voltages of the PTEG and WPTEG attached to a dry arm and a wet arm.

Figure S13 shows that the modified paper before superhydrophobic treatment rapidly absorbs water and curls up in contact with the solution and reaches saturation, while the modified paper after superhydrophobic treatment isolated the water from the device and maintains a the morphology and stiffness after 10 min, reflecting the waterproof and adaptability to humidity or water.

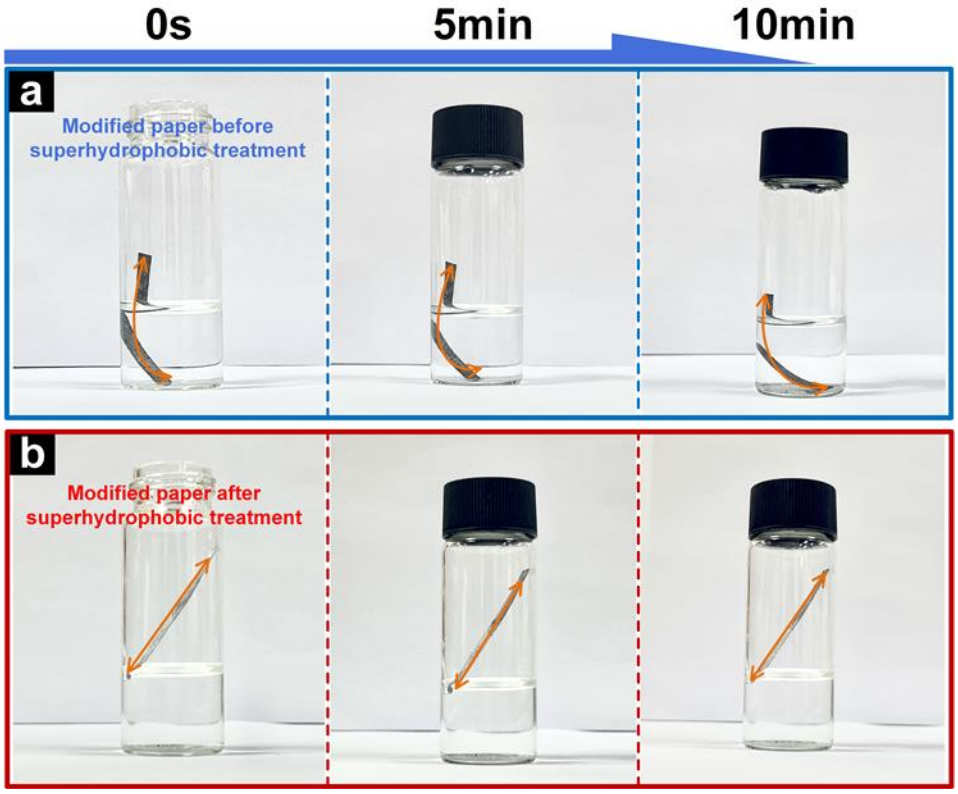

Figure S13. Optical images of modified paper (a) before and (b) after superhydrophobic treatment submerged in water.

Figure S14 shows that WPTEG has good flexibility.

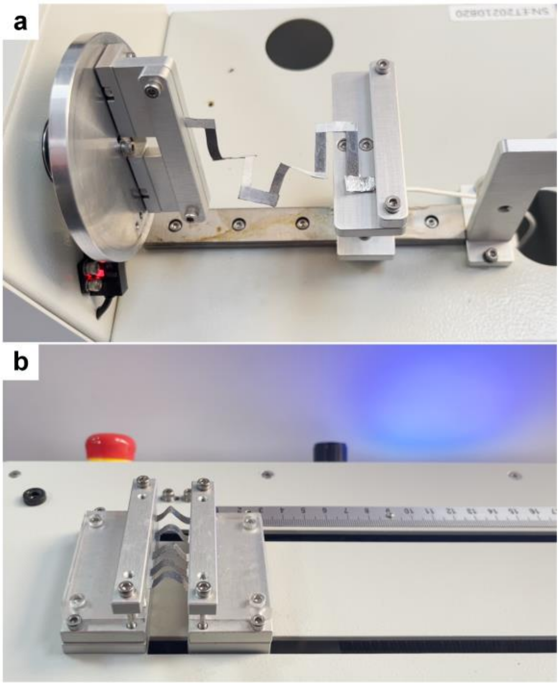

Figure S14. (a) Twisted WPTEG. (b) Curved WPTEG.

After 50 wet-dry cycles, the changes of Seebeck coefficient and internal resistance are less than 5% and 4% with the humidity reached ~100%, as shown in Figure S15. The slight changes in internal resistance and As shown in Figure S16(a), we stick the WPTEG to our wrist and insert it into cold water(25 degree, 40 seconds). The output voltage of the WPTEG is 4.94mV. Also, we stick WPTEG onto a hose filled with hot water(100℃) and immerse the hose deep into cold water. The output voltage of the WPTEG is 10.33mV, as shown in Figure S16(b). The results shows that the WPTEG is still able to harvest thermal energy from the human body, water pipes and other heat sources at underwater environments. Prior to application, the interface is tightly encapsulated so that the WPTEG is not affected by short circuits in water.

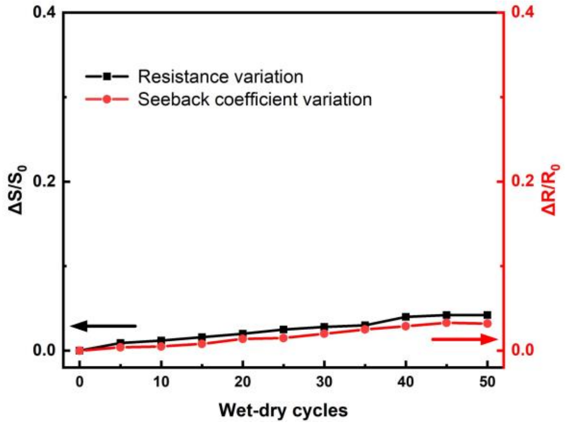

Figure S15. The material durability of WPTEG after 50 cycles.

Seebeck coefficient indicate that the modified paper has high stability, waterproofing, and durability.

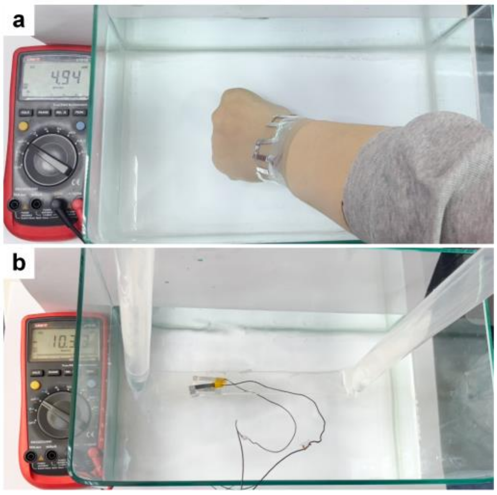

Figure S16. (a) Photograph of a WPTEG absorbing body heat from an underwater environment (25 degree, 40 seconds). (b) Photograph of a WPTEG absorbing thermal power from a hose filled with hot water (100℃) in an underwater environment.

References

1. Yuan, Z.; Tang, X.; Xu, Z.; Li, J.; Chen, W.; Liu, K.; Liu, Y.; Zhang, Z. Screen-printed Radial Structure Micro Radioisotope Thermoelectric Generator. *Appl. Energ.* **2018**, *225*, 746–754.
2. Kim, S. J.; Choi, H.; Kim, Y.; We, J. H.; Shin, J. S.; Lee, H. E.; Oh, M.-W.; Lee, K. J.; Cho, B. J. Post Ionized Defect Engineering of The Screen-printed $Bi_2Te_{2.7}Se_{0.3}$ Thick Film for High Performance Flexible Thermoelectric Generator. *Nano Energy* **2017**, *31*, 258–263.
3. Francioso, L.; De Pascali, C.; Farella, I.; Martucci, C.; Cretì, P.; Siciliano, P.; Perrone, A. Flexible Thermoelectric Generator for Ambient Assisted Living Wearable Biometric Sensors. *J. Power Sources* **2011**, *196*, 3239–3243.
